# Distinct Inhibitory Effects of Statin Lactones on Ca^2+^- and Na^+^/K^+^-ATPases

**DOI:** 10.3390/molecules31193423

**Published:** 2026-09-26

**Authors:** Carlos Cruz-Cortés, Jaroslava Šeflová, L. Michel Espinoza-Fonseca

**Affiliations:** 1 Department of Internal Medicine, Division of Cardiovascular Medicine, University of Michigan, Ann Arbor, MI 48109, USA; ccruzcor@med.umich.edu; 2 Center for Arrhythmia Research, University of Michigan, Ann Arbor, MI 48109, USA; 3 Department of Health and Biomedical Sciences, University of Texas Rio Grande Valley, Brownsville, TX 78521, USA; jaroslava.seflova@utrgv.edu

**Keywords:** P-type ATPases, SERCA, Na^+^/K^+^-ATPase, statins, structure–activity relationships, heterocycles

## Abstract

P-type ATPases share a conserved catalytic architecture but have different sensitivities to small-molecule inhibitors. We previously identified several statins as inhibitors of the sarco/endoplasmic reticulum Ca^2+^-ATPase (SERCA), but the extent to which this inhibitory activity reflects broader pharmacological cross-reactivity among P-type ATPases remains unknown. Here, we compared the effects of seven structurally diverse statin lactones, which share a common δ-lactone moiety but contain distinct core scaffolds, including hexahydronaphthalene-based and aromatic heterocyclic ring systems, on SERCA and the Na^+^/K^+^-ATPase (NKA). The structurally related lovastatin and simvastatin produced similar concentration-dependent inhibitory profiles toward SERCA and NKA, despite the otherwise distinct pharmacological sensitivities of these pumps. In contrast, atorvastatin, rosuvastatin, and pitavastatin showed more divergent inhibitory profiles, generally with greater inhibition of SERCA than NKA at higher concentrations. Pravastatin and fluvastatin, however, had minimal effects on either ATPase over the concentration range examined. These findings build on our previous identification of selected statins as SERCA inhibitors by demonstrating that their activity can extend to NKA. Overall, the results reveal substantial structure-dependent variation in the effects of statin lactones on these P-type ATPases, ranging from convergent inhibition to differential inhibition and minimal activity.

## 1. Introduction

P-type ATPases are a large family of membrane transport proteins that couple ATP hydrolysis to the active transport of ions, lipids, and organic molecules across biological membranes [1,2]. Among the best-characterized members are the sarco/endoplasmic reticulum Ca^2+^-ATPase (SERCA) and Na^+^/K^+^-ATPase (NKA), which mediate the transport of Ca^2+^ and Na^+^/K^+^, respectively [3,4]. Although these pumps differ substantially in ion specificity and transmembrane architecture, they share the characteristic catalytic organization of P-type ATPases, including conserved actuator, phosphorylation, and nucleotide-binding domains that coordinate ATP hydrolysis with the conformational transitions required for ion transport [5].

Despite their homology, SERCA and NKA generally have different sensitivities to small-molecule inhibitors. Classical inhibitors illustrate this distinction: thapsigargin selectively inhibits SERCA through a transmembrane binding site [4], whereas cardiotonic steroids (CTSs) target NKA through a structurally distinct extracellular binding region [6]. Our recent studies have provided an additional example of this selectivity: Ro 41-0960, a nitrocatechol-containing benzophenone, inhibits NKA but has substantially weaker activity toward SERCA [7]. In addition, N106, a small molecule that enhances SERCA2a function through increased SUMOylation, was recently found to partially inhibit NKA [8]. Thus, the conserved catalytic architecture of SERCA and NKA does not imply similar sensitivity to small-molecule inhibitors. Directly comparing the effects of the same compounds on SERCA and NKA therefore provides a useful means of establishing the extent to which inhibitor sensitivity is shared between these related ATPases.

Previous work from our group identified several statin lactones as inhibitors of SERCA, with several of these compounds producing partial inhibition of its ATPase activity [9]. Statins comprise a structurally diverse class of compounds that differ in their ring systems and substituents. In their lactone forms, the compounds share a six-membered δ-lactone ring but contain different molecular scaffolds, ranging from the hexahydronaphthalene-based structures of lovastatin, simvastatin, and pravastatin to the pyrrole, pyrimidine, indole, and quinoline heterocycles of atorvastatin, rosuvastatin, fluvastatin, and pitavastatin, respectively [10,11]. However, whether the inhibitory activity of statin lactones is restricted to SERCA or extends to other P-type ATPases has not been established.

Given the distinct inhibitor sensitivities of SERCA and NKA despite their shared catalytic machinery, comparison of the two ATPases can determine whether statin-mediated inhibition is selective for SERCA or extends to a homologous P-type ATPase. We therefore tested seven statin lactones for their effects on SERCA and NKA and directly compared their concentration-dependent inhibitory profiles. We show that statin lactones have a broad spectrum of effects on SERCA and NKA. Lovastatin and simvastatin produce similar concentration-dependent inhibition of the two ATPases, whereas atorvastatin, rosuvastatin, and pitavastatin show more divergent inhibitory profiles. In contrast, pravastatin and fluvastatin produce little inhibition of either ATPase over the concentration range examined. These findings reveal an unexpected convergence in the biochemical sensitivity of SERCA and NKA to selected statin lactones while demonstrating that ATPase inhibition varies among members of this compound class.

## 2. Results and Discussion

### 2.1. Structurally Similar Lovastatin and Simvastatin Exhibit Convergent Inhibition of SERCA and NKA

Lovastatin and its semisynthetic derivative simvastatin are closely related statins characterized by a common hexahydronaphthalene core and lactone-containing side chain (Figure 1A). Their structures differ only by an additional methyl substituent in the ester side chain of simvastatin, resulting in very similar molecular architectures. We therefore used this closely related pair to determine whether structurally similar statin lactones have comparable inhibitory behavior toward SERCA and NKA.

Consistent with our previous observations, lovastatin inhibited SERCA in a concentration-dependent manner (Figure 1B). Negligible changes in SERCA activity were observed at submicromolar concentrations, with greater inhibition becoming apparent as the lovastatin concentration increased. At 100 µM, approximately 50% of control SERCA activity remained. Interestingly, lovastatin produced a closely overlapping inhibitory profile toward NKA, with ATPase activity remaining near control levels at lower concentrations and declining at higher concentrations to a similar level of residual activity at 100 µM. No significant differences between SERCA and NKA activity were detected at any concentration (adjusted *p* > 0.05). Thus, under the experimental conditions used, lovastatin displayed similar concentration-dependent inhibitory behavior toward SERCA and NKA, with no evident preference for either ATPase.

Simvastatin produced a similar pattern of cross-inhibition, although inhibition became more pronounced at lower concentrations than with lovastatin (Figure 1C). SERCA and NKA activities remained near control levels at submicromolar concentrations and progressively decreased within the micromolar concentration range. At 100 µM, approximately 45–55% of control activity remained for both ATPases. As observed with lovastatin, the SERCA and NKA concentration-dependent profiles closely tracked one another across the concentration range, and no significant differences between the two ATPases were detected at any concentration (adjusted *p* > 0.05).

The close correspondence between SERCA and NKA inhibition by lovastatin and simvastatin is notable given the distinct pharmacological profiles of these ATPases. Common sensitivity of P-type ATPases to the same inhibitor can nevertheless occur, as illustrated by orthovanadate, a classical phosphate-analogue inhibitor of both SERCA and NKA [12,13]. Pharmacological cross-reactivity between SERCA and NKA has also been reported for structurally diverse organic compounds, including clotrimazole [14,15], ivermectin [16], and tetrahydrocarbazole derivatives [17]. These studies demonstrate that chemically distinct small molecules can inhibit both ATPases, although with compound-dependent differences in potency and mechanism. In this context, the closely matched concentration-dependent responses to lovastatin and simvastatin are particularly interesting, with both compounds exhibiting convergent inhibition of the two ATPases despite their modest structural difference.

Importantly, these observations do not establish that the shared structural features of lovastatin and simvastatin are responsible for their convergent inhibitory behavior. Moreover, inhibition of ATPase activity in these biochemical assays does not by itself establish direct binding of the statin lactones to SERCA or NKA, and the similar concentration-dependent inhibition of the two ATPases does not establish interaction with a conserved binding site or a common molecular mechanism. Rather, the present results demonstrate that two otherwise pharmacologically distinguishable P-type ATPases have similar sensitivity to these closely related statin lactones. Whether this relationship extends to other members of the statin class was therefore examined next.

### 2.2. Structurally Distinct Statins Exhibit Differential Inhibition of SERCA and NKA

We next determined whether the convergent inhibition observed with lovastatin and simvastatin extends to structurally distinct statin lactones by examining atorvastatin, rosuvastatin, and pitavastatin (Figure 2A). In contrast to the closely related hexahydronaphthalene-containing structures of lovastatin and simvastatin, these compounds contain distinct aromatic and heterocyclic scaffolds while retaining a common lactone-containing side chain.

Atorvastatin inhibited both SERCA and NKA in a concentration-dependent manner, but the responses of the two ATPases diverged as the concentration increased (Figure 2B). At lower concentrations, both enzymes retained activity relatively close to control levels, whereas SERCA activity declined more prominently than NKA activity at higher concentrations. At 100 µM, SERCA retained ~55% of control activity compared with ~76% for NKA, and this difference was significant (adjusted *p* = 0.041). Thus, unlike the closely overlapping profiles observed with lovastatin and simvastatin, atorvastatin produced greater inhibition of SERCA than NKA at higher concentrations.

Rosuvastatin produced a different inhibitory profile (Figure 2C). NKA activity remained largely preserved across the concentration range examined, whereas SERCA activity progressively declined at higher rosuvastatin concentrations, resulting in increasing separation between the two ATPases. The difference reached statistical significance at 50 µM, where SERCA retained ~73% of control activity compared with ~92% for NKA (adjusted *p* = 0.016). At 100 µM, SERCA activity decreased further to ~64%, whereas NKA activity remained largely preserved at ~94%. These results indicate greater sensitivity of SERCA than NKA to rosuvastatin under the conditions used in this study.

Pitavastatin inhibited both SERCA and NKA in a concentration-dependent manner (Figure 2D). Both ATPases remained relatively unaffected at lower concentrations, followed by progressively greater inhibition within the micromolar range. At intermediate-to-high concentrations, SERCA activity declined more prominently than NKA activity, with the largest difference observed at 50 µM, where SERCA retained ~43% of control activity compared with ~65% for NKA. However, no differences between SERCA and NKA activity reached statistical significance at any concentration (adjusted *p* > 0.05). At 100 µM, substantial inhibition of both ATPases was observed, with residual activities of ~43% for SERCA and ~52% for NKA. Thus, pitavastatin inhibited both ATPases, with more pronounced inhibition of SERCA at intermediate-to-high concentrations.

Taken together, these results demonstrate that the closely matched inhibition of SERCA and NKA observed with lovastatin and simvastatin is not uniformly retained among structurally distinct statin lactones. Atorvastatin and rosuvastatin showed greater inhibition of SERCA than NKA at higher concentrations, whereas pitavastatin substantially inhibited both ATPases, with more pronounced inhibition of SERCA at intermediate-to-high concentrations. Thus, the relative responses of SERCA and NKA vary considerably among individual statin lactones.

### 2.3. Pravastatin and Fluvastatin Have Minimal Effects on SERCA or NKA Activity

To further define the range of ATPase responses among statin lactones, we tested the effects of pravastatin and fluvastatin on SERCA and NKA (Figure 3A). Pravastatin retains a hexahydronaphthalene-based scaffold closely related to those of lovastatin and simvastatin, whereas fluvastatin possesses a substantially different indole-containing scaffold.

Pravastatin had minimal effects on either ATPase (Figure 3B). SERCA and NKA activities remained close to control levels throughout the concentration range, including at 100 µM, with no clear concentration-dependent inhibitory response. No significant differences between SERCA and NKA activity were detected at any concentration (adjusted *p* > 0.05). The lack of appreciable inhibition by pravastatin is particularly notable given its close structural relationship to lovastatin and simvastatin. Indeed, although all three compounds share a hexahydronaphthalene-based scaffold and lactone moiety, pravastatin did not reproduce the convergent inhibition observed with lovastatin and simvastatin. This suggests that the shared hexahydronaphthalene and lactone-containing architecture alone is insufficient to confer inhibition of these ATPases.

Fluvastatin similarly had minimal effects on SERCA or NKA ATPase activity (Figure 3C). The activities of both ATPases remained largely preserved across the concentration range, with no clear concentration-dependent inhibitory response. As with pravastatin, no significant differences between SERCA and NKA activity were detected at any concentration (adjusted *p* > 0.05). The minimal inhibitory effects of fluvastatin, despite its structurally distinct indole-containing scaffold, further illustrate the heterogeneous effects of statin lactones on these ATPases.

### 2.4. Qualitative Structure–Activity Relationships Among Statin Lactones

Comparison of the seven statin lactones revealed several qualitative relationships between molecular structure and ATPase inhibition. We examined the statins in their lactone forms because our previous work showed that the corresponding hydroxyacid forms did not appreciably inhibit SERCA [9]. Whether the hydroxyacid forms similarly lack inhibitory activity toward NKA remains to be determined. The most informative comparison is provided by lovastatin, simvastatin, and pravastatin, which share closely related hexahydronaphthalene-based scaffolds and a common lactone moiety [10,11] but have different inhibitory behavior. Lovastatin and simvastatin produced closely matched concentration-dependent inhibition of SERCA and NKA, whereas pravastatin had minimal effects on either ATPase. The relatively minor structural difference between lovastatin and simvastatin did not substantially alter their convergent inhibition of the two pumps. In contrast, pravastatin contains an additional hydroxyl substituent on the hexahydronaphthalene ring relative to lovastatin and simvastatin, increasing the polar substitution of this otherwise closely related scaffold [10,11]. Consistent with its greater polar substitution, pravastatin is substantially more hydrophilic than lovastatin and simvastatin [18]. The additional hydroxyl substitution is associated with reduced inhibitory activity toward both SERCA and NKA, indicating that the shared hexahydronaphthalene-based scaffold and lactone moiety are not sufficient for ATPase inhibition and that relatively modest structural modifications within this series can be associated with substantial differences in activity.

The heterocyclic statins showed a similarly diverse range of inhibitory profiles despite sharing the lactone functionality. Atorvastatin, rosuvastatin, fluvastatin, and pitavastatin contain pyrrole, pyrimidine, indole, and quinoline ring systems, respectively, resulting in substantial variation in their molecular scaffolds [10,11]. Atorvastatin, rosuvastatin, and pitavastatin each produced appreciable inhibition of SERCA and, to varying degrees, NKA, whereas fluvastatin had minimal effects on either ATPase. The retention of inhibitory activity across three substantially different heterocyclic scaffolds suggests that no single ring system is required for ATPase inhibition, while the minimal activity of fluvastatin further indicates that the presence of a heterocycle and lactone moiety alone is insufficient to confer inhibitory activity. The variable activities of these compounds indicate that differences in the heterocyclic scaffold and its substitution are associated with both the magnitude of inhibition and the relative effects on SERCA and NKA.

Overall, these comparisons indicate that ATPase inhibition by statin lactones is sensitive to structural features beyond the common lactone functionality or the presence of a particular core scaffold. The contrast among the closely related hexahydronaphthalene-containing compounds is particularly informative, showing that relatively modest differences in substitution can be associated with substantial changes in ATPase inhibition. The heterocyclic series complements this observation by showing that substantially different ring systems are associated with a broad range of activities, from appreciable inhibition of both ATPases to minimal activity. Although the limited number of compounds and absence of systematically varied analogues preclude establishment of a formal structure–activity relationship or quantitative QSAR model, the present results identify qualitative trends that provide a basis for future studies aimed at defining the structural determinants of SERCA and NKA inhibition by statin lactones. The inhibitory potencies of the statin lactones are also modest compared with those of classical high-affinity inhibitors of SERCA and NKA. Thapsigargin inhibits SERCA at subnanomolar concentrations [19], whereas ouabain inhibits NKA in the nanomolar range under appropriate assay conditions [20]. In contrast, appreciable inhibition by the statin lactones examined here generally occurred within the micromolar concentration range. Thus, these compounds should not be considered high-affinity ATPase inhibitors comparable to the established pharmacological inhibitors of SERCA or NKA. Whether statin lactones could serve as starting scaffolds for the development of P-type ATPase modulators remains uncertain. Their structural complexity and the absence of a systematically varied analogue series currently preclude definitive identification of the structural features responsible for ATPase inhibition. Defining these features may ultimately enable the design of simplified or optimized derivatives with improved potency and selectivity.

Several limitations should be considered when interpreting these findings. First, SERCA and NKA were assayed under different experimental conditions optimized for each enzyme, including differences in temperature and buffer composition. Because ATPase activities were normalized to the corresponding untreated controls measured under identical conditions, differences in basal enzymatic activity associated with these assay conditions are minimized in the relative comparisons presented here. Nevertheless, temperature and buffer composition could influence the inhibitory response to individual compounds, and the comparative profiles should therefore be interpreted in the context of the respective assay conditions. Second, the present study examined porcine SERCA2a and NKA α1, and the inhibitory profiles observed here may not necessarily extend quantitatively to other ATPase isoforms or species. Despite the high degree of conservation among P-type ATPases, isoform- and species-dependent differences can influence their pharmacological sensitivity. Evaluation of additional SERCA and NKA isoforms will therefore be necessary to establish the broader applicability of these observations. Third, the present findings are based on in vitro biochemical measurements of ATPase activity using isolated enzyme preparations. SERCA and NKA were obtained from different porcine tissues and prepared using different biochemical procedures, and differences in tissue-specific membrane composition and the biochemical environment retained after preparation could influence the inhibitory response to individual compounds. Consequently, the inhibitory profiles observed under these conditions may differ in cellular environments, where factors such as compound availability, membrane composition, and intracellular metabolism could influence the effects of statin lactones on SERCA and NKA. Accordingly, differences in tissue source and biochemical preparation should be considered when comparing the two ATPases, and the present results should not be assumed to reflect equivalent ATPase inhibition in cells or in vivo. Fourth, the concentrations producing appreciable ATPase inhibition in the present study are substantially higher than the circulating concentrations generally achieved during therapeutic statin use, which are typically in the nanomolar range [21]. Therefore, the inhibitory effects observed here should not be interpreted as evidence that comparable SERCA or NKA inhibition occurs at clinically relevant systemic exposures. Rather, the present experiments define the biochemical inhibitory profiles of the statin lactones under the conditions examined.

## 3. Materials and Methods

### 3.1. Chemicals

All chemicals used in this study were of reagent grade (purity > 95% by HPLC). Lovastatin lactone and simvastatin lactone were purchased from MedChemExpress (MCE; Monmouth Junction, NJ, USA); atorvastatin lactone and fluvastatin lactone from Santa Cruz Biotechnology (Dallas, TX, USA); and rosuvastatin lactone, pitavastatin lactone, and pravastatin lactone from Cayman Chemical (Ann Arbor, MI, USA).

### 3.2. SERCA Isolation

Cardiac SERCA (2a isoform) was isolated from porcine hearts obtained post-euthanasia from the University of Michigan Unit for Laboratory Animal Medicine. Ventricle free walls were minced and homogenized with a cold buffer that contained 9.1 mM NaHCO_3_, 0.9 mM Na_2_CO_3_, and a cocktail of protease inhibitors; the mixture was centrifuged at 6500× *g* for 30 min at 4 °C to remove debris. The supernatant was filtered, collected, and centrifuged at 14,000× *g* for 30 min at 4 °C. The collected filtrate was centrifuged at 47,000× *g* for 60 min at 4 °C. The pellet was resuspended in a solution containing 600 mM KCl and 20 mM Tris (pH 6.8). The suspension was centrifuged at 120,000× *g* for 60 min at 4 °C, and the pellet was resuspended in a solution containing 300 mM sucrose, 5 mM MOPS, and protease inhibitors (pH 7.4). The protein concentration of the SR microsomal fraction was determined using the Pierce Coomassie Plus assay kit (ThermoFisher Scientific, Waltham, MA, USA). The protein concentration was 3.4 µg/mL. The microsomal membranes were aliquoted, frozen in liquid nitrogen, and stored at −80 °C.

### 3.3. SERCA ATPase Activity Assays

We performed SERCA (isoform 2a) activity assays using an enzyme-coupled NADH-linked ATPase assay described previously by us [9]. We measured the activity of Ca^2+^ ATPase in μmol·min^−1^·mg^−1^ from the decrease in absorbance of NADH at 340 nm at 25 °C in a 96-well format using an H1 Synergy (BioTek, Winooski, VT, USA) microplate reader. Each well contained a 200 µl final volume of assay buffer containing SERCA buffer (50 mM MOPS, 100 mM KCl, 5 mM MgCl_2_, and 1 mM EGTA, pH 7), 5 U lactate dehydrogenase, 5 U pyruvate kinase, 1 mM phosphoenolpyruvate, 5 mM ATP, 0.2 mM NADH, 2 µg of microsomal suspension, 2 µM Ca^2+^ ionophore A23187, and a free Ca^2+^ concentration of 2.5 µM. Under control conditions in the absence of statin, the thapsigargin-sensitive SERCA ATPase activity was approximately 0.4 μmol·min^−1^·mg^−1^. Concentration-response curves were constructed using a nonlinear regression fit at compound concentrations of 0.1, 0.25, 0.5, 1, 2.5, 5, 10, 25, 50, and 100 µM. Prior to the ATPase assays, we incubated the small molecules for 30 min at 25 °C with the reaction mixture. ATP hydrolysis was initiated immediately upon ATP addition, and absorbance was monitored continuously at 1-min intervals for 60 min. For comparative inhibition analyses, specific SERCA ATPase activities were normalized to untreated control preparations to account for variability across independent preparations. Data were analyzed by nonlinear regression using GraphPad Prism 10 (GraphPad Software, Boston, MA, USA). Concentration–response data were fitted using a four-parameter logistic model with a variable slope. Data are reported as mean ± SEM (*N* = 3).

### 3.4. NKA Isolation

NKA (α1 isoform) was isolated from porcine kidney outer medulla using the established protocols [22,23,24], with minor modifications. Fresh porcine kidneys were obtained post-mortem from the University of Michigan Unit for Laboratory Animal Medicine. The outer medulla was dissected, mixed in a 1:1 (tissue:buffer) ratio with ice-cold buffer containing 250 mM sucrose and 30 mM L-histidine (pH 7.3), and incubated overnight at 4 °C. Tissue was subsequently minced and homogenized in ice-cold buffer containing 250 mM sucrose, 20 mM imidazole, and 1 mM EDTA (pH 7.4) using a glass-Teflon homogenizer. The homogenate was subjected to differential centrifugation at 3700× *g* for 20 min at 4 °C, followed by centrifugation of the supernatant at 7400× *g* for 20 min to remove cellular debris. Microsomal membranes were pelleted by ultracentrifugation at 38,000× *g* for 40 min. Enzyme preparations were aliquoted, flash-frozen in liquid nitrogen, and stored at −80 °C. Protein concentration was determined by the Bradford assay, and the protein concentration was 6.1 µg/mL. The presence of NKA α1 and the purity of the preparation were assessed by Western blotting, SDS-PAGE, and Coomassie staining. Functional integrity of purified NKA was confirmed by measuring ouabain-sensitive ATP hydrolysis prior to subsequent experiments.

### 3.5. NKA ATPase Activity Assays

NKA ATPase activity was determined as the activity sensitive to 500 μM ouabain (OUA) in μmol·min^−1^·mg^−1^ from the decrease in absorbance of NADH at 340 nm at 37 °C in a 96-well format using a Synergy H1 microplate reader (BioTek, Winooski, VT, USA). Each well contained a 200 μL final volume of assay buffer containing NKA buffer (30 mM MOPS, 130 mM NaCl, 20 mM KCl, and 4 mM MgCl_2_, pH 7.4), 5 U lactate dehydrogenase, 5 U pyruvate kinase, 1 mM phosphoenolpyruvate, 3 mM ATP, 0.2 mM NADH, and 0.8 μg of the purified NKA suspension. Under control conditions in the absence of statin, the ouabain-sensitive NKA ATPase activity was approximately 0.2 μmol·min^−1^·mg^−1^. In all cases, the residual activity in the presence of OUA was subtracted from the total estimated ATPase activity in OUA-untreated samples, and all data are presented as the OUA-sensitive ATPase activity. When applicable, IC_50_ values were calculated from the fitted concentration-response curve using GraphPad Prism 10 (Boston, MA, USA). Data are reported as mean ± SEM (*N* = 3).

### 3.6. Statistical Analysis

Data are presented as mean ± SEM from independent experiments. Statistical comparisons between SERCA and NKA activities were performed at each statin concentration using multiple unpaired *t*-tests with Welch’s correction, without assuming equal variances. The Holm–Šídák method was used to correct for multiple comparisons within each concentration-response series. This approach was selected because the primary statistical goal was to compare SERCA and NKA activities at each individual statin concentration rather than to evaluate overall effects across the complete concentration-response series. Adjusted *p*-values < 0.05 were considered statistically significant. Statistical analyses were performed using GraphPad Prism 10 (GraphPad Software, Boston, MA, USA).

## 4. Conclusions

In this study, we showed that statin lactones have different effects on SERCA and NKA, ranging from closely matched inhibition of both ATPases to differential inhibition and minimal effects on either pump. The structurally related lovastatin and simvastatin displayed remarkably similar concentration-dependent inhibitory profiles toward SERCA and NKA, despite the otherwise distinct pharmacological sensitivities of these ATPases. In contrast, the structurally distinct atorvastatin, rosuvastatin, and pitavastatin exhibited more divergent inhibitory profiles, generally with greater inhibition of SERCA than NKA at higher concentrations. Pravastatin and fluvastatin, however, produced little change in the activity of either ATPase over the concentration range examined. Thus, the extent and pattern of SERCA and NKA inhibition vary substantially among individual statin lactones.

These findings extend our previous identification of selected statins as SERCA inhibitors by demonstrating that their inhibitory activity can extend to NKA. The striking correspondence between SERCA and NKA inhibition by lovastatin and simvastatin contrasts with the differential effects of several structurally distinct statin lactones and the minimal activity of pravastatin and fluvastatin. The contrasting activities of the closely related lovastatin, simvastatin, and pravastatin further indicate that the shared hexahydronaphthalene-based scaffold and lactone moiety are not sufficient to confer ATPase inhibition. Together with the diverse inhibitory profiles of the heterocyclic statins, these observations reveal qualitative relationships between molecular structure and ATPase inhibition, indicating that activity depends on structural features beyond the common lactone functionality or the presence of a particular core scaffold. Future studies examining systematically varied analogues and additional P-type ATPases will help define the structural determinants of ATPase inhibition by statin lactones. Cellular studies will also be required to determine whether the inhibitory profiles observed in vitro are retained in a more physiologically relevant environment, while experiments addressing potential membrane-dependent or nonspecific effects will help establish the molecular basis and specificity of the observed inhibition.

## Figures and Tables

**Figure 1:**
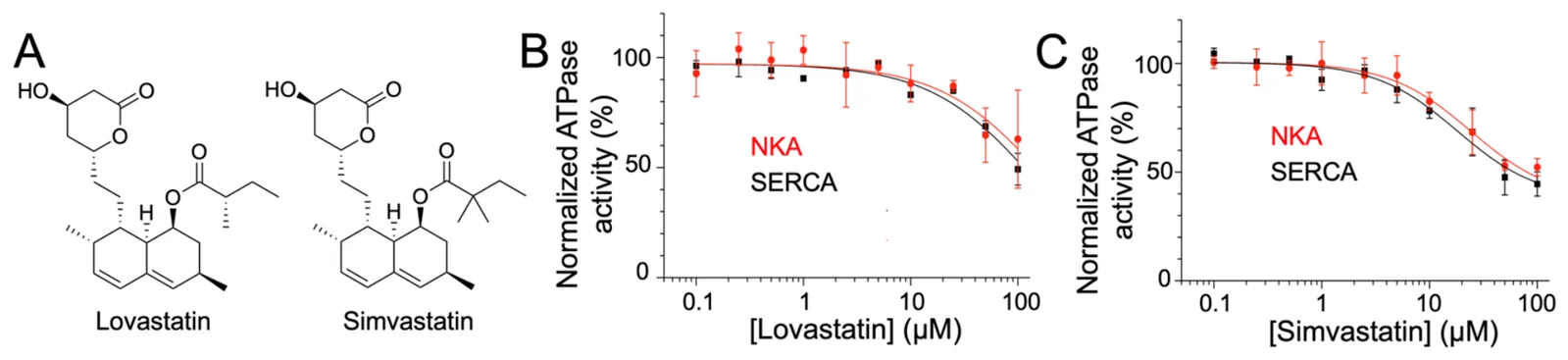
Lovastatin and simvastatin exhibit similar concentration-dependent inhibition of SERCA and NKA. (**A**) Chemical structures of lovastatin and simvastatin. (**B**,**C**) Concentration-dependent effects of lovastatin (**B**) and simvastatin (**C**) on SERCA (black squares) and NKA (red circles) ATPase activities. ATPase activities are expressed as percentages of the corresponding control activity in the absence of statin. Data are presented as mean ± SEM (*N* = 3). Curves represent nonlinear regression fits to the concentration-response data.

**Figure 2:**
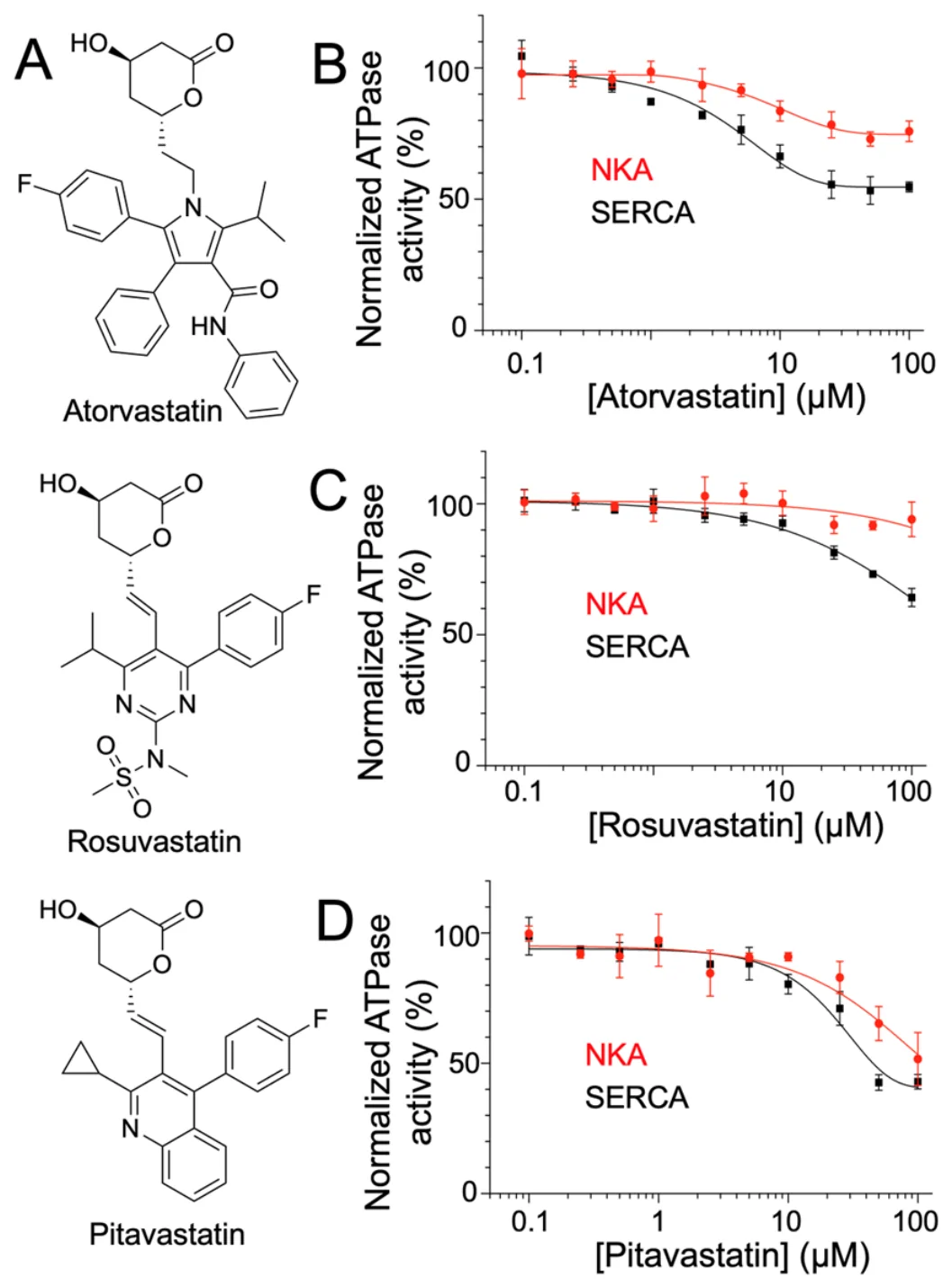
Structurally distinct statin lactones exhibit differential inhibition of SERCA and NKA. (**A**) Chemical structures of atorvastatin, rosuvastatin, and pitavastatin. (**B**–**D**) Concentration-dependent effects of atorvastatin (**B**), rosuvastatin (**C**), and pitavastatin (**D**) on SERCA (black squares) and NKA (red circles) ATPase activities. ATPase activities are expressed as percentages of the corresponding control activity in the absence of statin. Data are presented as mean ± SEM (*N* = 3). Curves represent nonlinear regression fits to the concentration-response data.

**Figure 3:**
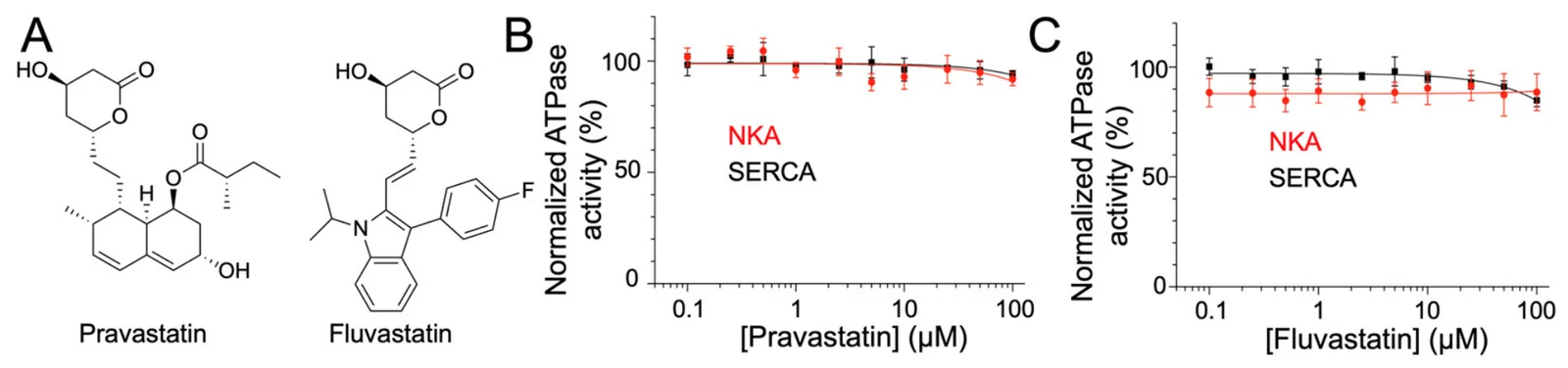
Pravastatin and fluvastatin have minimal effects on SERCA and NKA ATPase activities. (**A**) Chemical structures of pravastatin and fluvastatin. (**B**,**C**) Concentration-dependent effects of pravastatin (**B**) and fluvastatin (**C**) on SERCA (black squares) and NKA (red circles) ATPase activities. ATPase activities are expressed as percentages of the corresponding control activity in the absence of statin. Data are presented as mean ± SEM (*N* = 3). Curves represent nonlinear regression fits to the concentration-response data.

## Data Availability

All the data are reported in the manuscript.
